# Immunogenic cell death-associated biomarkers classification predicts prognosis and immunotherapy efficacy in pancreatic ductal adenocarcinoma

**DOI:** 10.3389/fonc.2023.1178966

**Published:** 2023-03-30

**Authors:** Wenguang Peng, Jiarui Yang, Long Xia, Xiangjun Qian, Guojie Long, Hao Zhang, Jiancong Xie, Junzhang Zhao, Lei Zhang, Weidong Pan

**Affiliations:** ^1^ Department of Pancreatic-Hepato-Biliary-Surgery, The Sixth Affiliated Hospital, Sun Yat-Sen University, Guangzhou, Guangdong, China; ^2^ Department of Hepatobiliary-Pancreatic-Splenic Surgery, Inner Mongolia Autonomous Region People’s Hospital, Hohhot, China; ^3^ Department of Gastroenterology, Guangdong Provincial Key Laboratory of Colorectal and Pelvic Floor Disease, The Sixth Affiliated Hospital of Sun Yat-Sen University, Guangzhou, Guangdong, China

**Keywords:** immunogenic cell death, pancreatic ductal adenocarcinoma, risk model, prognosis, tumor immune microenvironment

## Abstract

**Introduction:**

Immunogenic cell death (ICD) is a sort of regulated cell death (RCD) sufficient to trigger an adaptive immunological response. According to the current findings, ICD has the capacity to alter the tumor immune microenvironment by generating danger signals or damage-associated molecular patterns (DAMPs), which may contribute in immunotherapy. It would be beneficial to develop ICD-related biomarkers that classify individuals depending on how well they respond to ICD immunotherapy.

**Methods and results:**

We used consensus clustering to identify two ICD-related groupings. The ICD-high subtype was associated with favorable clinical outcomes, significant immune cell infiltration, and powerful immune response signaling activity. In addition, we developed and validated an ICD-related prognostic model for PDAC survival based on the tumor immune microenvironment. We also collected clinical and pathological data from 48 patients with PDAC, and patients with high EIF2A expression had a poor prognosis. Finally, based on ICD signatures, we developed a novel PDAC categorization method. This categorization had significant clinical implications for determining prognosis and immunotherapy.

**Conclusion:**

Our work emphasizes the connections between ICD subtype variations and alterations in the immune tumor microenvironment in PDAC. These findings may help the immune therapy-based therapies for patients with PDAC. We also created and validated an ICD-related prognostic signature, which had a substantial impact on estimating patients' overall survival times (OS).

## Introduction

The immune system is crucial for tumor monitoring and prevention as it plays a part in preventing carcinogenesis and obstructing its growth (1). The concept of immunogenic cell death (ICD) is brought up about chemotherapy for cancer. It is a unique cell death method that can provide a particular immunity against the fine cell antigens found in tumors. This immune response will cause the number of T lymphocytes in tumor tissues to grow following chemotherapy. After chemotherapy, the prognosis of the cancer is strongly correlated with the quantity of T cells. Damage-associated molecular patterns (DAMPs) might be released by dying tumor cells due to ICD (2). High mobility group box protein B1 (HMOB1), adenosine triphosphate (ATP), heat shock protein (HSP), high mobility group box protein B1 (HMGB1), type I interferon (IFNI), and annexin 1 (ANXA1) are a few of the more significant DAMPs. They have the capacity to mobilize antigen-presenting cells (APCs) and activate T cells to elicit adaptive immune responses against tumor antigens. Then, tumor-specific immune responses may be triggered, directly destroying tumor cells or triggering anti-tumor immunity, increasing the long-term efficacy of anticancer drugs (3, 4). Due to its immunogenicity, the immune system’s activation in tumors, and the production of several tumor antigens, ICD is anticipated to offer fresh concepts and approaches for anti-tumor immunotherapy (5). Therefore, patients should be investigated further in a clinical setting to evaluate the likelihood of ICD. It would be beneficial to identify biomarkers that categorize individuals based on how likely they are to respond to immunotherapy for ICD.

Pancreatic cancer is an aggressive malignancy in humans with a 5-year survival rate of only 5% (6). About 90% of pancreatic cancers are ductal adenocarcinomas that arise from the ductal epithelium, known as pancreatic ductal adenocarcinoma (PDAC). Currently, radiation, conventional chemotherapy, and surgical resection are the mainstays of pancreatic cancer treatment. Although radical resection remains the preferred course of action for pancreatic cancer, the postoperative recurrence rate can reach 85%, and more than 80% of patients cannot have surgical resection because of local or distant metastases (7). Nevertheless, radiotherapy, particularly stereotactic body radiation therapy (SBRT), and chemotherapy, such as gemcitabine monotherapy or combined with albumin-bound paclitaxel and the FOLFIRINOX regimen (5-fluorouracil, leucovorin, irinotecan, and oxaliplatin), have limited clinical benefits for patients with advanced pancreatic cancer and can only marginally improve their condition and prognosis. The tumor microenvironment (TME) is the main mediator of tumor development and resistance to drug therapy or immune checkpoint inhibitors (ICIs). It plays a vital role in pancreatic cancer’s occurrence, growth, and metastasis, but its exact mechanism is unknown. Immunological cells and elements of the cell matrix make up the majority of the pancreatic cancer immune microenvironment. Among them, the immune cells mainly include T cells such as CD4+ and CD8+, B lymphocytes, related tertiary lymphoid structures (TLS), and a large number of immunosuppressive cells such as T regulatory cells (T regulatory cells), Treg cells, myeloid-derived suppressor cells (MDSCs) and tumor-associated macrophages (TAMs) (8). Immunotherapy has become an increasingly essential treatment method for pancreatic cancer in recent years as TME research has grown (9). The sustainable development of cancer immunotherapy, a better understanding of T cell responses to therapies targeting immune checkpoints, and the efficacy of clinical studies of drugs that block related immune checkpoints will lead to an increase in the number of studies predicting and identifying accurate biomarkers for PDAC immunotherapy.

This research intended to uncover ICD-associated biomarkers and construct an ICD risk model to predict the immune microenvironment and PDAC prognosis. In the future, this method can assist physicians in making better treatment decisions.

## Materials and methods

### Data collection

For the training set, RNA-seq transcriptome information and matched clinicopathology data of 176 PDAC patients were obtained from TCGA (https://portal.gdc.cancer.gov/). Patients’ data for the validation set were obtained from ICGC (https://dcc.icgc.org/). In order to verify the effect of related genes on the prognosis of PDAC patients, a total of 48 consecutive patients who underwent radical resection for pancreatic ductal adenocarcinoma (PDAC) at the Inner Mongolia Autonomous Region People’s Hospital from January 1, 2015 to December 31, 2015 were collected in this study.

### Consensus clustering

By using agglomerative pam clustering with 1-Pearson correlation distances and resampling 80% of the samples for ten iterations, ConsensusClusterPlus (Wilkerson and Hayes, 2010, ConsensusClusterPlus: a class discovery tool with confidence assessments and item tracking, DOI:10.1093/bioinformatics/btq170) was used to perform the cluster analysis. The empirical cumulative distribution function plot calculated the ideal number of clusters.

### Reverse transcription quantitative PCR

48 PDAC specimens were received from Department of Hepatobiliary-Pancreatic-Splenic Surgery, Inner Mongolia Autonomous Region People’s Hospital (Hohhot, China) and utilized to verify EIF2A expression in PDAC. The RT-PCR method has been described in our previous study (10). GAPDH served as the EIF2A control.

### Identification of differentially expressed genes

The Limma package (version: 3.40.2) of R software was used to analyze the differential mRNA expression. The modified P values were looked at to correct TCGA results that were falsely positive. The threshold for mRNAs with differential expression was set at |fold change| >2 and adjusted P< 0.05.

### Functional enrichment analysis

The differences in signal pathways and biological effects between the ICD low and high cohorts were compared using Gene Ontology (GO) and Kyoto Encyclopedia of Genes and Genomes (KEGG) studies. To assess GO and KEGG pathways, the R software’s “clusterProfiler” tool (11) and SangerBox (http://sangerbox.com/) (12) were used. The q-value and p-value criteria of <0.05 were used as the foundation for GO and KEGG enrichment analysis.

### Gene set enrichment analysis

The enrichment of the MSigDB Collection was evaluated using GSEA to determine whether there were significant differences in the set of genes expressed between the ICD low and high cohorts (c2.cp.kegg.v7.4.symbols.gmt). SangerBox (12) was used to conduct the analysis.

### Immune landscape characterization between two ICD subgroups

The expression data of 176 PDAC samples were put into CIBERSORT (HTTPS://cibersort.stanford.edu/), which was then used to calculate the relative percentage of 22 immune cell types (13). The results are shown in a landscape map after we compared the relative percentage of 22 immune cell types across the two ICD groupings.

### Prediction of immunotherapy effectiveness

Analyses of tumor immune dysfunction and exclusion (TIDE) have been carried out to assess the efficacy of immunotherapy. An analytical method called TIDE (http://tide.dfci.harvard.edu/) allows for the prediction of the immunotherapy response utilizing two key tumor immune evasion mechanisms: T cell malfunction and T cell infiltration that is suppressed in tumors with low CTL levels.

### Analysis of somatic mutations

The TCGA GDC Data Portal provided somatic mutation information for the PDAC samples in “maf” format. The “Maftools” program in R software was then used to create waterfall graphs, which made it easier to see and summarize the altered genes.

### Survival evaluation

Using the survminer and survival packages in R, a Kaplan-Meier (KM) analysis was performed to compare the overall survival (OS) between the low and high ICD risk cohorts. The multivariate Cox analysis was used to determine if the risk score is an independent risk factor for OS in PDAC, whereas the univariate Cox analysis was used to identify the prospective prognostic indicators.

### Construction of risk signatures related to ICDs

In order to determine the precise coefficient values of each detected relationship, a LASSO cox regression analysis was used for the immune-associated genes that were shown to be statistically significant in the univariate Cox regression study. A common regression analysis technique called LASSO combines variable selection and regularization to enhance the prediction capability and interpretability of the resulting statistical model.

## Results

### Two ICD-associated subtypes were identified *via* consensus clustering

**Figure 1:**
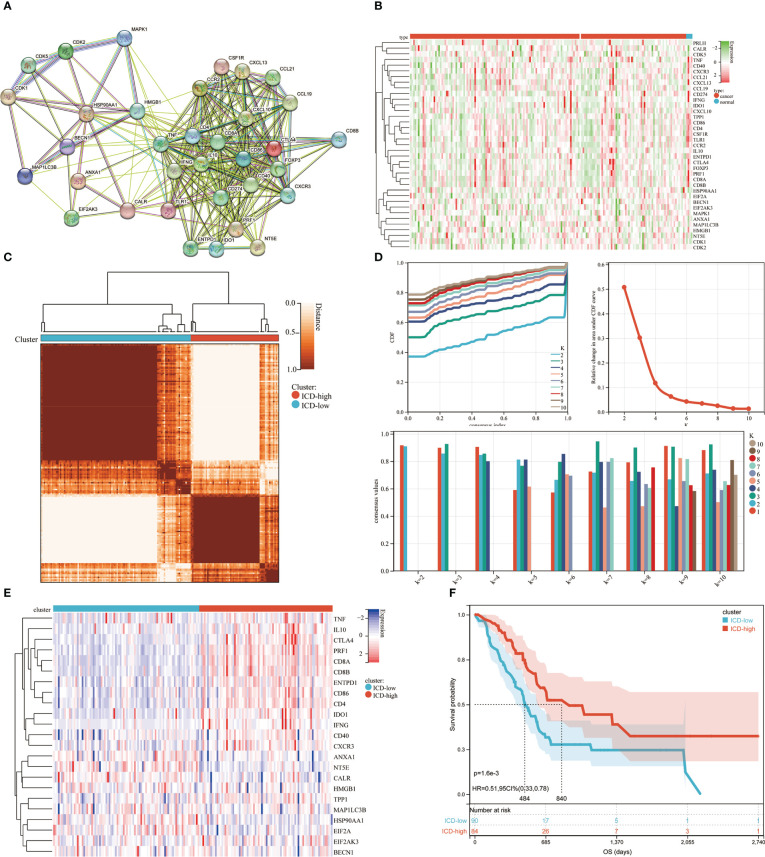
Consensus clustering for the identification of subtypes related to ICD. **(A)** PPI (Protein-protein interactions) among the genes related to ICD; **(B)** The TCGA database heatmap displays 37 ICD gene expression patterns in normal and PDAC samples; **(C)** The consensus clustering solution (k = 2) for 23 genes in 176 PDAC samples is represented by a heatmap; **(D)** Consensus clustering’s delta area curve showed the change in area under the cumulative distribution function (CDF) curve for k = 2 to 10; **(E)** Heatmap of 37 ICD-associated genes’ expression levels across various subtypes. Blue symbolized low expression, whereas red denoted high expression; **(F)** ICD-high and ICD-low subtype OS Kaplan-Meier curves.

A comprehensive literature review that discovered the ICD-related genes earlier was published by Chiaravalli (4). To further illuminate the relationships between these ICD-related genes, we carried out protein-protein interaction (PPI) network analysis using the STRING database (**Figure 1A**). We also examined the ICD gene expression patterns in samples from normal and PDAC (**Figure 1B**). Next, we used consensus clustering to identify the PDAC clusters connected to ICDs. Following k-means clustering, two clusters with distinct ICD gene expression patterns were found in the TCGA cohort (**Figures 1C, D**). ICD-related gene expression levels were generally high in cluster C2, indicating an ICD-high subtype. Instead, cluster C1 showed low expression levels consistent with an ICD-low subtype (**Figure 1E**). We classified clusters C1 as an ICD-low subtype and C2 as an ICD-high subtype. Additionally, survival analyses showed that these ICD-based subcategories had varying clinical outcomes. The ICD-low subtype generally had a poor prognosis, while the ICD-high subtype was linked to favorable clinical results (**Figure 1F**).

### Differentially expressed genes and signal pathways in various ICD subtypes

**Figure 2:**
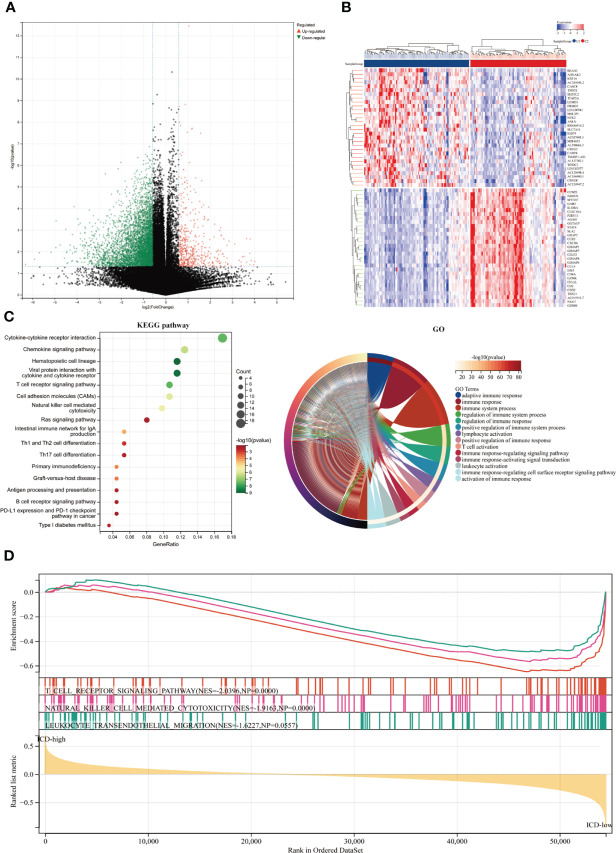
Identifying differentially expressed genes (DEGs) and underlying signaling pathways in various subtypes. **(A)** The volcano figure depicted the distribution of DEGs measured between ICD-high and ICD-low subgroups in the TCGA cohort, with a threshold of |log2 Fold change| > 1 and P 0.05. **(B)** The heatmap depicted the expression of DEG in various subtypes. **(C)** The KEGG and GO signaling pathway enrichment analyses were shown by dot and circle plots. The dot size reflected gene count, and the dot color denoted - log10 (p. adjust-value); **(D)** GSEA analysis identified the fundamental signal pathway that differentiated ICD-high and ICD-low subgroups.

We identified the major DEGs and signal pathways in each subtype to understand the molecular mechanism in the modulation of prognosis since the ICD high subtype presented with favorable clinical outcomes and the ICD low subtype presented with a poor prognosis. We discovered a total of 527 dysregulated genes here (**Figures 2A, B**). Among them, 23 genes were up-regulated in expression. AC132186.1, AC091173.1 and AC068228.2 were top up-regulated DEGs. A total of 496 genes expressing downregulated. GZMAP1, CD300LG and AL162457.1 were top down-regulated DEGs. The upregulated genes in the ICD high subtype were enriched in immune-related activities like cytokine and cytokine receptor interaction, chemokine signaling pathway, hematopoietic cell lineage, viral protein interaction with cytokine and cytokine receptor, T cell receptor signaling pathway, cell adhesion molecules, natural killer cell-mediated cytotoxicity, and Ras signaling pathway, among others (**Figure 2C**). These findings suggested a link between the immune-active microenvironment and the ICD high subtype. By assessing the ICD high and low groups, we used GSEA to further identify the relevant signaling pathways engaged in the ICD high subgroup. Gene sets associated with immunological pathways, such as the T cell receptor signaling pathway, natural killer cell-mediated cytotoxicity, and leukocyte trans-endothelial migration were differentially enriched in the ICD groups (**Figure 2D**).

### The landscape of the tumor microenvironment and somatic mutations in ICD-high and ICD-low subtypes

**Figure 3:**
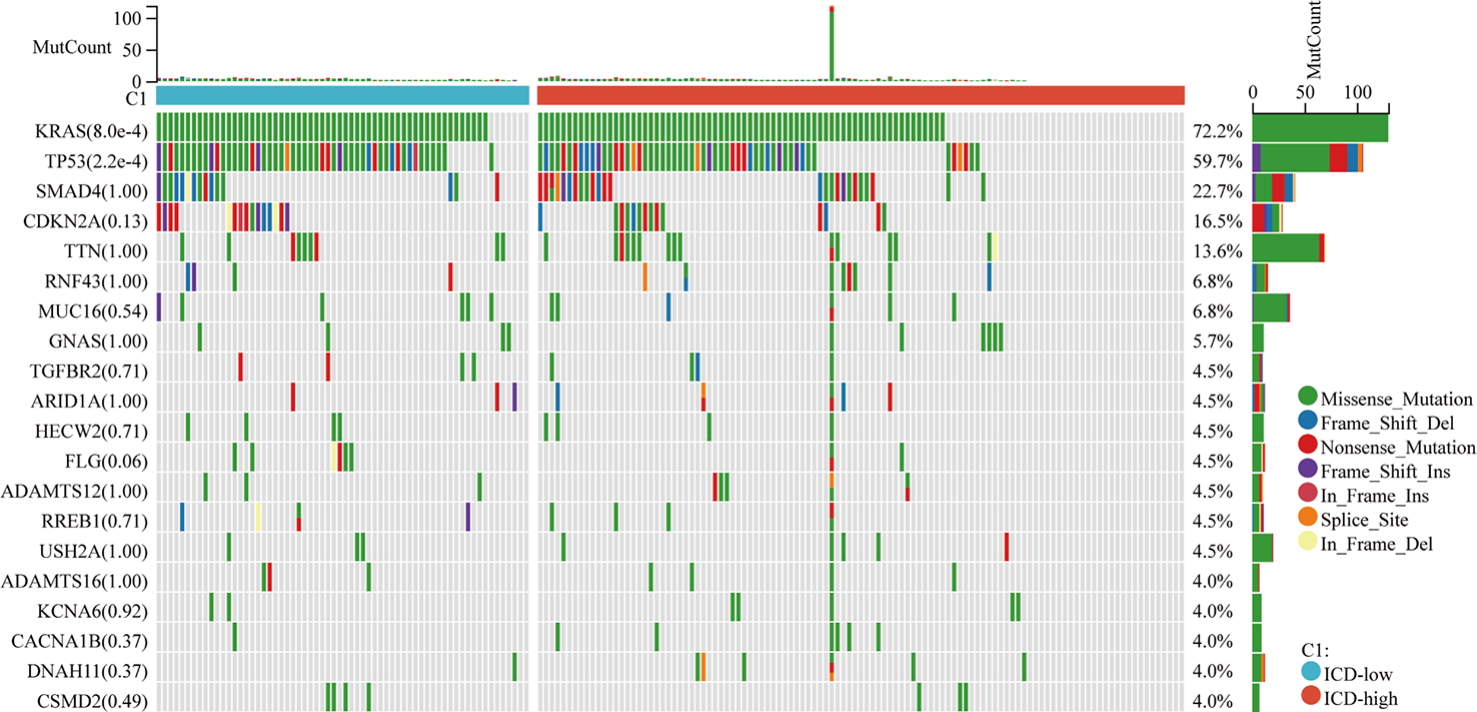
Somatic mutations in distinct ICD classes are investigated. The waterfall map showed the top ten most often altered genes in ICD-high and ICD-low subtypes. A total of 175 samples with identified mutations were included, with mapping samples including 156 (89.1%).

Among these subgroups, we identified distinctive somatic mutation patterns (**Figure 3**). Although the most frequent mutations were KRAS, TP53, SMAD4, CDKN2A, and TTN, the proportional frequency varied among the various subtypes. KRAS and TP53 mutations were more common in ICD-low subtypes than in ICD-high subtypes, accounting for 86.4 percent and 71.4 percent of the total, respectively, compared to just 72.2 percent and 59.7 percent (p < 0.001).

**Figure 4:**
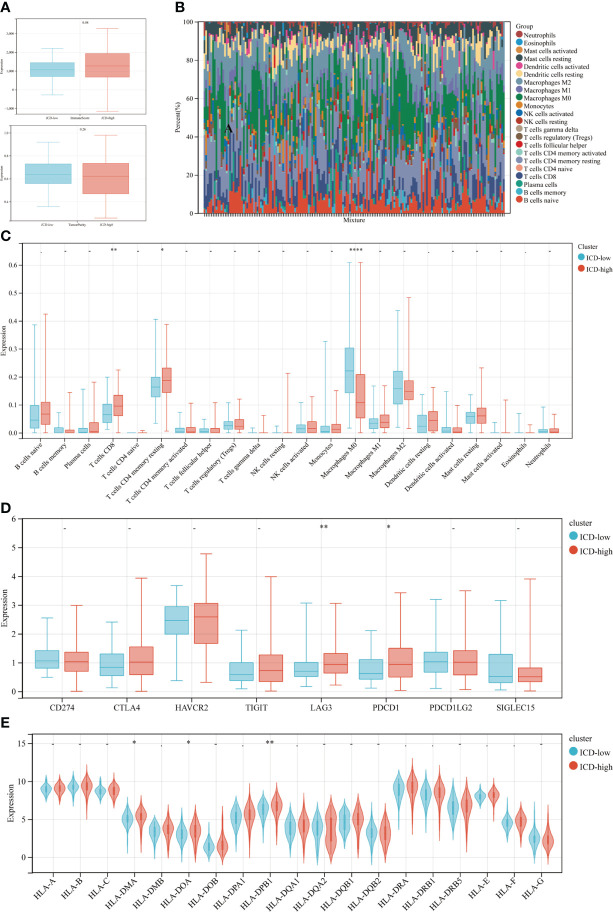
Immune landscape of ICD-low and ICD-high subtypes. **(A)** The median and quartile estimates for each immunological score and tumor purity score are shown in box plots. **(B)** Immune infiltration percentage in ICD-high and ICD-low subgroups; **(C)** The violin plot depicted considerably diverse immune cells across distinct subtypes. **(D, E)** Box and violin plots showed the expression of numerous immunological checkpoints **(D)** and HLA genes **(E)** differing between ICD-high and ICD-low subgroups. *P < 0.05, **P < 0.01, and ****P < 0.0001.

ICD may significantly affect the activation of several anticancer immune responses, according to growing data. This study examined the differences in the tumor microenvironment between two subtypes. Overall, the immunological score was greater and the tumor purity was lower in the ICD-high subtype compared to the ICD-low subtype (**Figure 4A**), however, the differences were not significant (p=0.08, p=0.26, respectively). The LM22 signature matrix and the CIBERSORT method were then used to compare the immunological infiltration of 22 different immune cell types between two subtypes. The findings from 176 PDAC patients in the TCGA were summarized in **Figure 4B**. Exceptionally high percentages of CD8 T cells and resting CD4 T cell memory were seen in individuals with the ICD-high subtype with low rates of macrophage M0 (**Figure 4C**). In addition, the ICD-high subtype showed upregulation of some of the human leukocyte antigen (HLA) genes and immunological checkpoints. On the other hand, the ICD-low subtype showed the reverse tendency (**Figures 4D, E**). These revealed a relationship between the immune-hot phenotype and the ICD-high subtype and a link between the immune-cold phenotype and the ICD-low subtype.

### ICD risk signature development and validation

**Figure 5:**
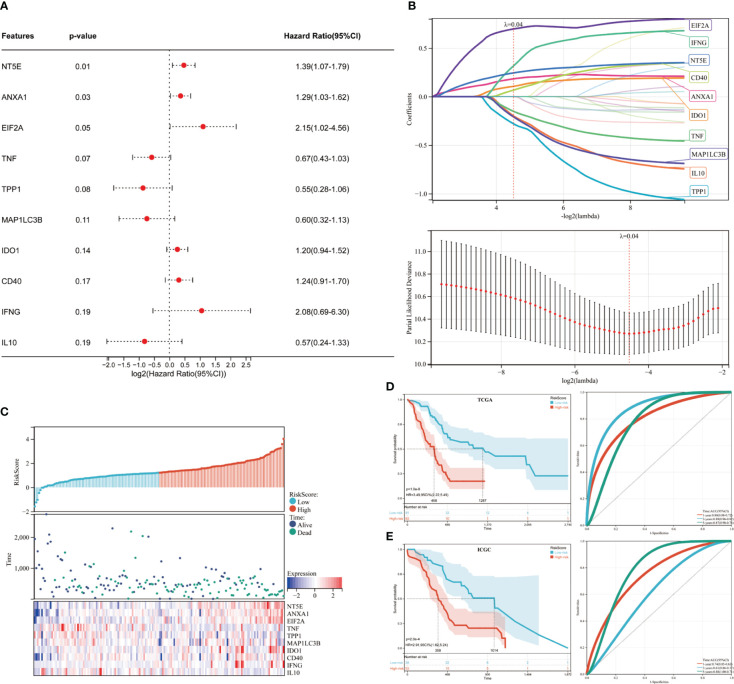
Developing and validating the ICD risk signature. **(A)** Multivariate Cox analysis assesses the prognostic value of ICD genes in terms of OS; **(B)** Lasso Cox analysis identified ten genes most associated with OS in the TCGA dataset; **(C)** Distribution of risk scores, survival status of each patient, and heatmaps of prognostic 10-gene signature in the TCGA database; **(D, E)** Kaplan-Meier analyses demonstrate the prognostic significance of the risk model in the TCGA and ICGC cohort.

**Figure 6:**
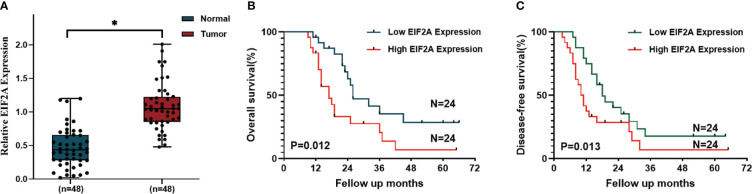
The correlation between EIF2A expression and prognosis of PDAC patients. **(A)** The relative amount of EIF2A expression in PDAC tissues and normal pancreatic tissues as determined by RT-qPCR. **(B, C)** Kaplan–Meier curves of OS and DFS in EIF2A-high and EIF2A-low subtypes. *P < 0.05.

We then developed a prognosis model based on genes related to ICD. The Cox multivariate analysis revealed that 3 ICD-related genes were strongly associated with patients’ OS (**Figure 5A**). The LASSO regression analysis investigated and identified 10 ICD-related genes for the prediction model (**Figure 5B**). The following algorithm serves as the foundation for the risk-score model: Risk rating=(0.1835)*ANXA1+(-0.1996)*IL10+(0.1059)*IDO1+(0.0669)*CD40+(-0.1518)*TNF+(0.3163)*IFNG+(-0.2802)*TPP1+(0.2445)*NT5E+(-0.2086)*MAP1LC3B+(0.6976)*EIF2A. Furthermore, we focused on the link between survival status and risk score. Our findings revealed that the number of alive statuses in the low-risk group was much higher than in the high-risk cohort (**Figure 5C**). KM analysis was used to further establish the prognostic value of this risk profile in PDAC (**Figure 5D**). A high-risk score was discovered to correlate with poor OS in the TCGA cohort, further supported by equivalent outcomes in the ICGC cohort (**Figure 5E**). To verify the relationship between ICD-related gene EIF2A and prognosis, we collected data of 48 PDAC patients from Inner Mongolia Autonomous Region People’s Hospital, of whom 24 were EIF2A high expression and 24 were low expression. The correlation between EIF2A expression and clinicopathologic characteristics of PDAC patients was shown in **Table 1**. EIF2A expression in PDAC tissues and normal pancreatic tissues was examined using qRT-PCR as a possible risk factor. EIF2A expression was shown to be higher in PDAC tissues when compared to normal pancreatic tissues (**Figure 6A**). High-expression of EIF2A was discovered to correlate with poor OS and DFS in the these patients (**Figures 6B, C**).

**Table 1:** Correlation between EIF2A expression and clinicopathologic characteristics of PDAC patients^a^.

Variable	Total	High expression of EIF2A	Low expression of EIF2A	*P* value
Total case	48	24	24	
**Age (year)**				0.082
≤60	23	8	15	
>60	25	16	9	
**Gender**				0.999
Male	29	14	15	
Female	19	10	9	
**CA-199**				0.667
≥37	42	22	20	
<37	6	2	4	
**Pathology Grade**				0.046*
Well	11	2	9	
Moderately	25	14	11	
Poor	12	8	4	
**T Stage**				0.470
T1	19	10	9	
T2	17	7	10	
T3	7	3	4	
T4	5	4	1	
**LN metastasis**				0.002*
N0	25	6	19	
N (N1&N2)	23	18	5	
**TNM Stage(AJCC^b^)**				0.038*
I (IA&IB)	18	6	12	
II (IIA&IIB)	22	11	11	
III	8	7	1	

a*:Chi-square(and Fisher’s exact) test, *P value <0.05.

b: American Joint Committee on Cancer (AJCC), patients were staged in accordance with the 8th Edition of the AJCC Cancer’s TNM Classification.

c: Bold values: There were significant differences between the two groups.

### The relationship between tumor microenvironment and ICD risk signature

**Figure 7:**
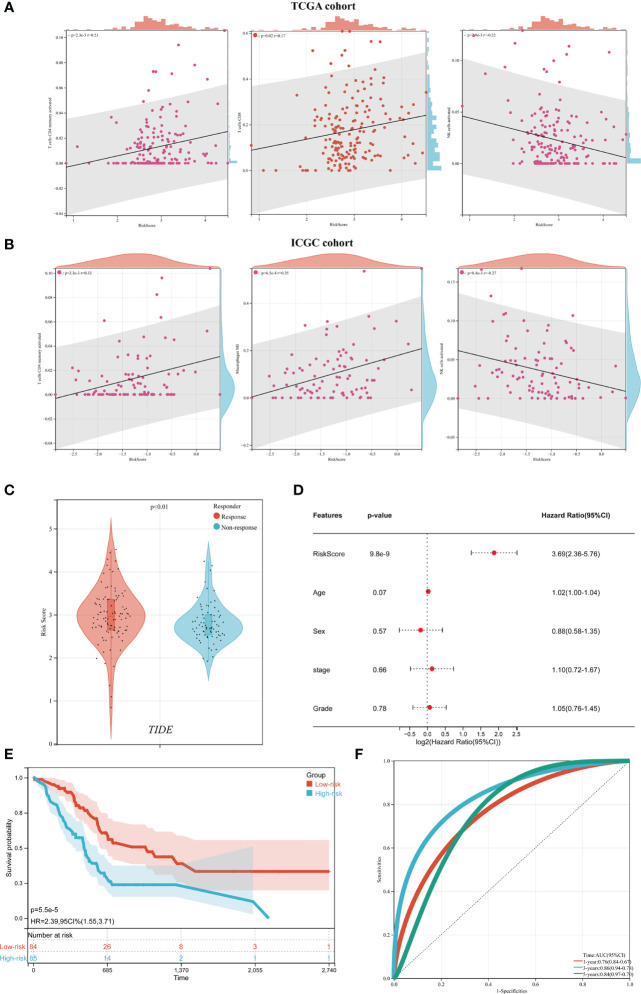
The relationship between the tumor microenvironment and the ICD risk profile. **(A, B)** Scatter plots demonstrate the relationship between risk score and activated CD4 memory cells, T cells CD8 and activated NK cells infiltration **(A)**, which was further supported by the ICGC cohort **(B)**; **(C)** The violin plot depicted the relationship between ICD risk score and immunotherapy response. **(D)** multivariate Cox analyses assessed the independent prognostic value of the ICD risk signature in PDAC patients. **(E, F)** Survival analysis was used to construct a risk model in the TCGA dataset **(E)** and ROC curve **(F)**.

Given that ICD plays essential biological functions in antitumor immune responses, the relationship between ICD risk score and the tumor microenvironment was carefully investigated. The findings revealed that whereas CD8 and activated CD4 memory cells showed an undesirable link in people with higher risk scores, activated NK cells showed a positive correlation in those with higher risk scores (**Figure 7A**). These findings were confirmed by the ICGC cohort (**Figure 7B**).

The predictive significance of the ICD risk signature in the prospective therapeutic effectiveness of immunotherapy was then assessed using TIDE. According to our findings, patients with low ICD risk scores may benefit more from immunotherapy since their ICD risk scores were greater in the group that had no response to immunotherapy (**Figure 7C**).

To assess the independent prognostic significance of the ICD risk signature, multivariate Cox analyses were undertaken. The multivariate study demonstrated that the ICD risk score might be an independent prognostic factor for patients withPDAC (**Figure 7D**). Based on these results, the AUCs for 1-, 3-, and 5-year overall survival were 0.76, 0.86, and 0.84, respectively (**Figure 7D**).

Based on the TCGA cohort, we also discovered that patients with low-risk scores tended to have much longer overall survival than patients with high-risk scores (**Figure 7E**). Additionally, the ICGC cohort showed similar AUCs and prognostic differences (**Figure 7F**).

## Discussion

Since chemotherapy is now the only approved treatment, pancreatic ductal adenocarcinoma (PDAC) is widely recognized for its poor prognosis, poor response to chemotherapy, and resistance to immunotherapy. In fact, it is characterized by an immunosuppressive TME and a robust desmoplastic reaction, which not only physically obstructs the delivery of drugs to the tumor site but also significantly impacts the homeostasis and aggressiveness of the tumor, interacting strongly with pancreatic cancer cells (PCC). Immunotherapy has drawn increasing interest because traditional therapies for chemotherapy were unable to significantly improve the overall survival rates of PDAC patients. Due to the distinct tumor microenvironment and limited cancer immunogenicity of PDAC, it is still not the breakthrough cure (14). It has been suggested that a fresh approach involves reshaping the TME by inducing innate and adaptive antitumor immunity (15). The concept of “immunogenic cell death” was defined as a particular sort of “regulated cell death” that can release “danger signals” or “DAMPs” to cause complete antigen-specific adaptive immune responses (16). For the treatment of cancer, the combination of immunogenic therapy and innovative immunotherapeutic regimens offers considerable potential (17). Therefore, it may be helpful to discover ICD-related biomarkers contributing to differentiating PDAC patients based on the advantages they experience after immunotherapy. We have shown that the expression of genes related to ICD is highly correlated with the tumor microenvironment and prognosis of PDAC. By consensus clustering based on the expression of ICD-related genes, we discovered two ICD subgroups. Positive clinical outcomes and a high degree of immune cell infiltration were linked to the ICD high subtype. Using 10 chosen ICD-related genes, we also developed and validated a predictive risk signature that divided PDAC patients into high- and low-risk groups. Additionally, this risk profile demonstrated a high level of OS predictive value and may serve as an independent prognostic predictor for patients with PDAC.

Chiaravalli et al. (4) have previously detailed the genes associated with ICD that were examined in our investigation. In summary, ICD parameters were found by doing a thorough literature search (using Scopus, Web of Knowledge, and PubMed for relevant research studies conducted *in vitro* using primary human immune cells and/or *in vivo* using mice). The final results revealed that the survival of patients with PDAC was correlated with 37 ICD-related genes. In our investigation, 10 of the 37 ICD-related genes—including ANXA1, IL10, IDO1, CD40, TNF, IFNG, TPP1, NT5E, MAP1LC3B, and EIF2A—were significantly associated with the prognosis of PDAC patients. Recent research revealed that when exposed to diverse microenvironmental stimuli during tumorigenesis, cancer cells suffered reduced canonical translation and switched their translational machinery to EIF2A-dependent translation (18). Dai et al (19) identified the pertinent PUM1 molecular mechanism in PDAC. PUM1 knockdown stimulated the PERK/EIF2/ATF4 signaling pathway in PDAC cells, inhibiting cell growth, invasion, and metastasis and promoting apoptosis.

Some chemical drugs, radiotherapy, photodynamic therapy, lysing virus, and other therapies act on tumor cells, causing endoplasmic reticulum (ER) stress and the production of reactive oxygen species (ROS), as well as releasing immune signaling molecules from the cell, increasing tumor immunogenicity and enhancing the recognition and presentation ability of dendritic cells (DCs), activating tumor-specific cytotoxic T cells (cytotoxic T cells), and stimulating the release of IL-2, IL This mechanism results in apoptosis, which is known as ICD in cancers (20). When a tumor develops ICD, a series of immune signal molecules are upregulated on the cell membrane’s surface and recognized by some receptors, promoting the synthesis and release of immune effectors and inducing the body’s immune response to kill the tumor cells; these immune signal molecules are known as DAMPs (21). These include endoplasmic reticulum calcium reticulum protein (CRT) exposed early on the cytosolic surface, extracellularly secreted adenosine triphosphate (ATP), heat shock protein (HSP)-antigen peptide complexes, and high mobility group protein B1 (HMGB1) released late (22). DAMPs and recognition receptors secreted extracellularly are required for the expression of ICD activity, promote DC maturation and nuclear factor-κB (NF-κB) activation through IFN-regulatory factors as well as MAPK and Akt pathways, facilitate migration and proliferation in local lymph nodes, enhance tumor antigen uptake and presentation, and produce IFN-γ, perforin-1, and granzyme B to trigger a direct cytotoxic response to kill residual tumor cells. Our study used consensual clustering to split the ICD sample into two categories in line with this findings. It was stated that the ICD-high subgroup had an immune-cold phenotype while the ICD-high subgroup had an immune-hot phenotype.

In this work, the risk model was verified in a different dataset and demonstrated appropriate survival prediction. Although this model is effective in predicting the prognosis of PDAC, the current study has a number of drawbacks. First, because the model was constructed utilizing a public database, we should proceed with caution when extending our findings to local patients. Second, additional research on the *in vivo* and *in vitro* functions and regulatory processes of these ten genes is required. Further prospective trials are also required to confirm the treatment implications of this risk model.

## Conclusion

Our findings demonstrate the relationship between ICD subtype variations and changes in the immunological tumor microenvironment in PDAC. These findings could help with immunotherapy-based PDAC treatment. We also developed and validated a predictive signature linked to ICD, which has a significant impact on forecasting patients’ overall survival time (OS).

## Data availability statement

The original contributions presented in the study are included in the article/supplementary material. Further inquiries can be directed to the corresponding authors.

## Author contributions

JZ, LZ and WDP designed and guided the study. WGP, JR and LX analyzed the data and wrote the manuscript. JX, GL, HZ, and JX drew the figures and conducted the experiments. All authors read and approved the final manuscript. All authors contributed to the article and approved the submitted version.
